# Atypical Presentation of Choroidal Folds: Steroid-induced Central Serous Chorioretinopathy-like Maculopathy

**DOI:** 10.4274/tjo.galenos.2019.92255

**Published:** 2019-12-31

**Authors:** Cumali Değirmenci, Filiz Afrashi, Serhad Nalçacı, Serap Bilge Çeper

**Affiliations:** 1 Ege University Faculty of Medicine, Department of Ophthalmology, İzmir, Turkey

**Keywords:** Corticosteroid, choroidal folds, rheumatoid arthritis, central serous chorioretinopathy-like maculopathy

## Abstract

This article reports a case of choroidal folds and central serous chorioretinopathy-like maculopathy induced by corticosteroid treatment. The patient was a 70-year-old woman who presented with decreased visual acuity in the right eye. She had a history of rheumatoid arthritis and was prescribed 20 mg leflunomide and 16 mg corticosteroid daily. Fundoscopy indicated bilateral macular edema and the presence of choroidal folds. Retinal imaging supported choroidal folds and central serous chorioretinopathy-like maculopathy. Corticosteroid therapy was discontinued, and the patient was followed up. Complete regression of the maculopathy was observed at 8-month follow-up examination.

## Introduction

Choroidal folds, or chorioretinal folds, are defined as undulations of the retina and choroid. Anatomically, they include the inner choroid, Bruch’s membrane, and the retinal pigment epithelium (RPE). On fundus examination, they are seen mostly outside the macular area and appear as pigmentary changes with yellow and dark streaks. Occasionally RPE atrophy occurs, which angiographically resembles an angioid streak.^1^^,2^

Known causes of choroidal folds are retrobulbar masses, thyroid eye disease, posterior scleritis, acquired hypermetropia, uveal effusion syndrome, ocular surgery, hypotony, and optic neuropathy.^2^,^^^3^ Cases with unknown etiology are defined as idiopathic. Visual acuity can be affected if the folds involve the macula. Although the pathogenic mechanism of choroidal folds is still unclear, two different theories have been suggested. The first theory postulates that the pathogenicity of the folds results from a close relationship between the choriocapillaris and Bruch’s membrane such that choroidal expansion can cause folds in Bruch’s membrane. The second theory suggests that the relationship between stress and strain that arises from the sclera and choroid may cause choroidal folds.^1^

The aim of this study is to present clinical data obtained at the first visit and follow up examination of a patient presenting with choroidal folds, central serous retinopathy (CSR)-like maculopathy, and hypermetropia.

## Case Report

**Figure 1:**
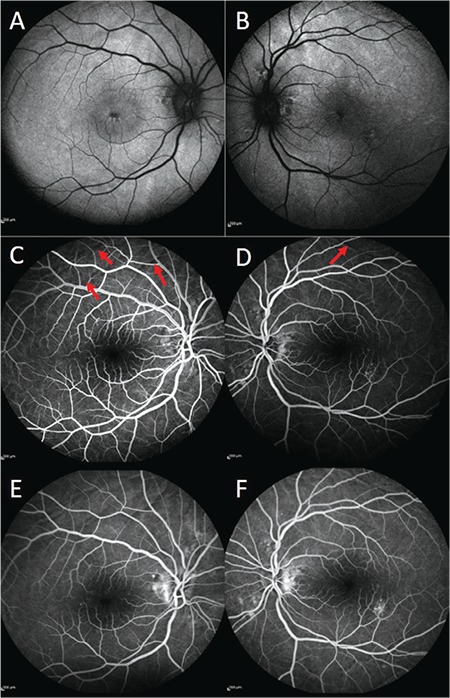
A,B) Fundus autofluorescence of the patient at initial examination showed central hypoautofluorescence related to macular edema; C,D) early phase of fluorescein angiography demonstrated choroidal folds (red arrows); E,F) late phase of fluorescein angiography showed points of leakage in the peripapillary and left inferotemporal areas

**Figure 2:**
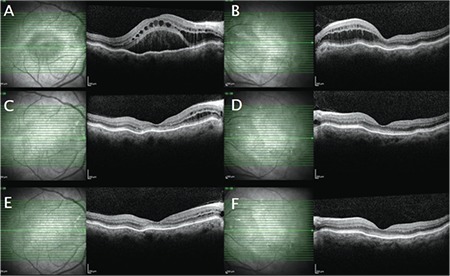
Optical coherence tomography at initial examination showed significant intraretinal and subretinal fluid; C, D) significant regression was observed at 4 months; E, F) complete regression was observed at 8 months

**Figure 3:**
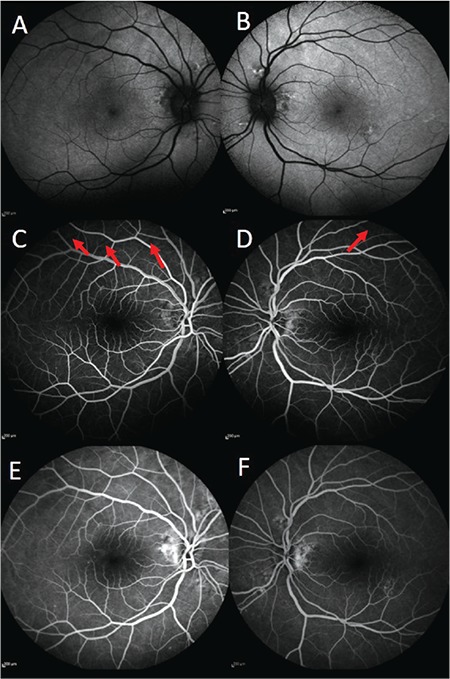
Fundus autofluorescence at 8 months showed regression of central hypoautofluorescence; C, D) early phase of fluorescein angiography demonstrated persistence of the choroidal folds (red arrows); E, F) late phase of fluorescein angiography showed no points of leakage

A 70-year-old woman presented with decreased visual acuity in the right eye that started 4 days earlier. She had undergone cataract surgery 4 years ago and had a history of rheumatoid arthritis and hypertension. She had been prescribed oral 20 mg leflunomide (Arava, Sanofi Sağlık Ürünleri, İstanbul) and 16 mg corticosteroid (Prednol, Mustafa Nevzat İlaç, İstanbul) daily 1.5 months earlier for rheumatoid arthritis. The patient underwent a complete ophthalmic examination. Her best corrected visual acuity (BCVA) was 0.2 in the right eye (+7.00 -2.00 α70) and 0.5 (+5.00 -2.50 α100) in the left eye. Intraocular pressure (IOP) measured with Goldman applanation tonometer was 18 mmHg in the right eye and 19 mmHg in left eye. Anterior segment examination showed posterior chamber intraocular lenses. Fundoscopy indicated bilateral macular edema up to the optic nerve and the presence of choroidal folds. Fluorescein angiography demonstrated leakage in the peripapillary region and fundus autofluorescence showed hyperfluorescence in the foveal area (Figure 1). Optical coherence tomography (OCT) revealed intraretinal and subretinal fluid accumulation in the right eye and intraretinal fluid accumulation in the left eye. Additionally, OCT showed the presence of chorioretinal undulation in both eyes (Figure 2). Corticosteroid therapy was discontinued and the patient was followed up. The patient’s axial length was measured as 19.26 mm in the right eye and 20.01 mm in the left eye. BCVA, IOP, anterior and posterior segment examination, and OCT were carried out in the follow-up period. At the 8 month examination, BCVA was 0.7 in the right eye and 0.6 in the left eye, IOP was within normal limits (14 mmHg bilaterally), the anterior segment was unchanged, and the posterior segment showed RPE changes in the macular area and peripheral retina in addition to the chorioretinal folds. OCT indicated the absence of intraretinal or subretinal fluid bilaterally. Fluorescein angiography and fundus autofluorescence findings indicated the regression of pathology (Figure 3).

## Discussion

Choroidal folds are defined as wrinkles of the RPE and choroid, and in the neural retina secondary to these. Choroidal folds should be carefully investigated clinically because of the underlying pathology. They are associated with inflammatory, neoplastic, and infectious diseases.^1,2^ Visual acuity, fundus examination, OCT, fluorescein angiography, indocyanine green angiography, ophthalmic ultrasonography, and magnetic resonance imaging are the tools that have diagnostic importance. In the present study, a 70-year-old female presented with choroidal folds, axial hypermetropia, and CSR-like maculopathy induced by corticosteroid use.

Olsen et al.^2^ defined three angiographic stages for choroidal folds. Stage 1 was described as alternating bands of hyperfluorescence and hypofluorescence, stage 2 as a breakdown of RPE along with the breaks in the Bruch’s membrane, and stage 3 as occult choroidal neovascularization. RPE atrophy may be seen in the valleys of these bands.^4^ In the present study, we observed the presence of alternating bands due to pigmentary changes in the chorioretinal folds and angioid streak-like lesions around the optic disc (Figure 2, 3). Therefore, we thought that the patient may have stage 2 choroidal folds.

The etiology of choroidal folds can be manifold. One of the most important etiologic causes is hypermetropia. In reporting the demographic characteristics of their patient group, Olsen et al.^2^ indicated that 18 of 25 patients with known etiology had hypermetropia. Saoji et al.^3^ presented a case with axial hypermetropia, choroidal folds, and subretinal fluid. The patient had short axial length and therefore was at risk of disease development.

Choroidal folds are generally not associated with subretinal or intraretinal fluid, with very few studies reporting this. One mechanism suggested for the accumulation of subretinal or intraretinal fluid is localized capillary and venous congestion along the folds leading to capillary hyperpermeability. It has also been suggested that choroidal folds can affect the function of the RPE and cause fluid accumulation.^3^^,5,6,7^ Our patient had CSR-like maculopathy and was taking systemic corticosteroids concurrently for the previous 2 months. Corticosteroid treatment may have contributed to the development of maculopathy in our patient, who already had choroidal congestion due to the presence of chorioretinal folds.

The relationship between corticosteroid intake and CSR is well defined. CSR is characterized by neurosensory retinal detachment and subretinal fluid accumulation. Although intraretinal fluid accumulation may occur, particularly in chronic cases, it is not typical for all cases. In previous reports, intraretinal and/or subretinal fluid accumulation with choroidal folds was annotated as CSR-like maculopathy or chorioretinal folds-related maculopathy. There are only a few case reports about this condition in the literature.^3^^,6,7,8^ In the present study, the patient was observed to have choroidal folds and a short axial length. Therefore, the patient was already at risk for developing CSR-like maculopathy, which may have been exacerbated by corticosteroid treatment. To our knowledge, this is the first report of a patient who had CSR-like maculopathy with choroidal folds who was also concurrently treated with corticosteroids.

We present a patient with choroidal folds and atypical retinal findings that resembled CSR. The etiology for the development of choroidal folds should be closely investigated. In the present case, the patient had short axial length, rheumatoid arthritis, and was taking corticosteroids, which most likely contributed to the pathology. The clinician should therefore be aware of additional systemic diseases and the corresponding treatments, as these may contribute to the etiology in atypical cases.
